# Evaluating the Effects of Various Antioxidants on Dentinal Tubule Penetrability of a Resin-Based Sealer: A Confocal Laser Microscopic Study

**DOI:** 10.7759/cureus.97436

**Published:** 2025-11-21

**Authors:** Sanjeev Srivastava, Shijita Sinha, Abhishek Singh, Aditya Singh, Pragyan Paliwal, Syed H Mehdii

**Affiliations:** 1 Department of Conservative Dentistry and Endodontics, Sardar Patel Post Graduate Institute of Dental & Medical Sciences, Lucknow, IND

**Keywords:** antioxidants, cashew nut shell liquid, confocal laser microscopy, lycopene, proanthocyanidin, rhodamine b dye, root canal sealer, sealer penetration, sodium hypochlorite

## Abstract

Background

This study aimed to evaluate the effects of various antioxidants on the dentinal tubule penetrability of a resin-based sealer using confocal laser microscopy.

Methodology

A total of 60 extracted human teeth were decoronated and prepared using Protaper Gold rotary files. The canals were irrigated sequentially with 3% sodium hypochlorite, 17% ethylenediaminetetraacetic acid, and distilled water. Samples were randomly divided into the following four groups (n = 15) based on the antioxidant used: Group 1 - control (no antioxidant), Group 2 - 6.5% proanthocyanidin, Group 3 - 34% cashew nut shell liquid, and Group 4 - 10% lycopene. Randomization was performed using a computer-generated random sequence to ensure unbiased specimen allocation. Following irrigation and drying, canals were obturated using AH Plus resin sealer mixed with Rhodamine B dye. Transverse sections were then examined under a confocal laser scanning microscope to assess sealer penetration. Data were statistically analyzed using one-way analysis of variance and Kruskal-Wallis tests.

Results

Group 4 (10% lycopene) demonstrated the greatest dentinal tubule penetration (359.00 ± 1.73 µm), followed by Group 2 (6.5% proanthocyanidin) (287.86 ± 1.55 µm) and Group 3 (34% cashew nut shell liquid) (261.00 ± 1.85 µm). The control group (Group 1) exhibited the least penetration (230.00 ± 1.92 µm). The differences among the groups were statistically significant (p < 0.05).

Conclusion

This study provides new evidence that antioxidant application enhances resin sealer penetration, potentially improving long-term root canal sealing efficacy. Within the limitations of this in vitro study, 10% lycopene exhibited the highest dentinal tubule penetrability of resin-based sealer, whereas the control group without antioxidant treatment showed the lowest. Antioxidant application may enhance resin sealer penetration and potentially improve the bonding interface.

## Introduction

Successful root canal treatment depends on accurate diagnosis, optimal access cavity preparation, thorough cleaning and shaping of the root canal system, effective debris removal, three-dimensional obturation of the canals, and appropriate coronal restoration. However, complete elimination of microorganisms remains difficult due to the intricate root canal anatomy, which includes apical deltas, isthmuses, and lateral canals [1]. Achieving a sterile canal environment, therefore, necessitates the use of effective endodontic irrigants.

Sodium hypochlorite (NaOCl) is the most widely used irrigating solution owing to its excellent tissue-dissolving and antimicrobial properties. Nonetheless, NaOCl can adversely affect the polymerization of resin-based sealers. The residual oxygen released as a byproduct during its reaction with water may remain entrapped within dentinal tubules, leading to reduced bond strength and compromised adhesion between resin sealers and dentin [2].

To overcome these limitations, the application of antioxidant or cross-linking agents has been advocated. These agents neutralize residual free radicals, thereby enhancing sealer penetration and improving bonding. Additionally, they inhibit matrix metalloproteinases responsible for collagen degradation in the hybrid layer, thus minimizing the risk of bond failure between resin and dentin [3].

In this study, three antioxidants, namely, lycopene, proanthocyanidin, and cashew nut shell liquid (CNSL), were evaluated for their influence on the dentinal tubule penetration of resin-based sealers. Proanthocyanidins are natural antioxidants capable of neutralizing oxygen free radicals and reducing oxidative stress and inflammation. CNSL, rich in anacardic acids, cardanol, and cardols, exhibits antimicrobial, anticancer, and anti-inflammatory properties and may function as an effective free-radical scavenger in endodontic applications [4]. Lycopene, a carotenoid abundant in tomatoes, is another potent antioxidant known to alleviate oxidative stress, modulate inflammation, and enhance metabolic function [5].

Accordingly, the objective of this study was to assess and compare the effects of lycopene, proanthocyanidin, and CNSL on the dentinal tubule penetrability of resin-based root canal sealers using confocal laser scanning microscopy (CLSM). The results aim to clarify the potential role of antioxidants in improving sealer penetration and thereby enhancing the long-term success of root canal treatment.

Despite numerous studies on endodontic irrigants, limited data exist on the comparative effects of natural antioxidants on sealer penetration. This study addresses that gap using standardized experimental protocols and confocal laser microscopy to evaluate the efficacy of three novel antioxidants.

## Materials and methods

A total of 60 freshly extracted single-rooted mandibular premolars, obtained for orthodontic or periodontal reasons, were selected for this study. Teeth exhibiting caries, cracks, restorations, resorptive defects, or calcifications were excluded. All specimens were thoroughly cleaned of soft tissue remnants and sterilized in an autoclave at 121°C and 15 psi for 15 minutes, then stored in distilled water at room temperature until further use to prevent dehydration.

Each tooth was decoronated at the cementoenamel junction using a diamond disc under water cooling to obtain a standardized root length of 14 mm from the apex. Biomechanical preparation was performed using ProTaper Gold rotary files up to size F2 (25/0.08) according to the manufacturer’s instructions. During instrumentation, canal irrigation was performed with 3 mL of 3% NaOCl after each file, followed by a final rinse with 3 mL of 17% ethylenediaminetetraacetic acid for one minute to remove the smear layer. A final flush with 5 mL of distilled water was performed to eliminate any residual irrigants.

The specimens were randomly divided into four groups (n = 15) based on the antioxidant solution used as a pretreatment agent, which was applied for one minute. Randomization was conducted using a computer-generated random sequence in Microsoft Excel, and each sample was assigned a unique identification code to ensure unbiased allocation and equal representation across groups (Table 1).

**Table 1:** Group distribution.

Group number	Group description
Group 1	Control group: no antioxidant was used
Group 2	Pretreated with 6.5% proanthocyanidin
Group 3	Pretreated with 34% cashew nut shell liquid
Group 4	Pretreated with 10% lycopene

Following antioxidant pretreatment, all canals were rinsed with distilled water and dried using sterile paper points. The canals were then obturated with ProTaper F2 gutta-percha cones and AH Plus resin sealer mixed with 0.1% Rhodamine B dye to facilitate fluorescent visualization under CLSM. The specimens were stored at 37°C in 100% humidity for 24 hours to ensure complete sealer polymerization.

Calibration and microscopic analysis

Before data collection, the confocal laser scanning microscope was calibrated using a standard fluorescent reference slide to ensure measurement accuracy. All imaging parameters, including laser intensity, pinhole aperture, and detector gain, were standardized across specimens to maintain consistency.

Each sample was sectioned longitudinally in the buccolingual direction using a diamond-coated carborundum disc under continuous distilled water irrigation to prevent heat generation or artifact formation. The sections were observed under CLSM at 10× magnification to assess the depth of resin sealer penetration into the dentinal tubules. Measurements were recorded at the standardized point (apical third) using CLSM software.

Statistical analysis

All measurements were tabulated, and the mean sealer penetration depth (in µm) was calculated for each group. Data were analyzed using one-way analysis of variance (ANOVA) and the Kruskal-Wallis test, with statistical significance set at a p-value <0.05. Statistical analysis was performed using SPSS software version 26.0 (IBM Corp., Armonk, NY, USA).

## Results

The mean values and standard deviations of dentinal tubule penetration depth for all groups are presented in Table 2. Among the tested groups, the highest mean depth of sealer penetration was observed in group 4 (10% lycopene) (359.00 ± 1.73 μm), followed by group 2 (6.5% proanthocyanidin) (287.86 ± 1.55 μm) and group 3 (CNSL) (261.00 ± 1.85 μm). The lowest mean penetration was recorded in group 1 (control, no antioxidant) (230.00 ± 1.92 μm).

**Table 2:** Mean depth of dentinal tubule penetration (μm) of resin-based sealer among different antioxidant groups. Values are expressed as mean ± standard deviation (SD). Statistical analysis was performed using one-way analysis of variance and Tukey’s post hoc test (p < 0.05 considered significant).

Group description	Mean ± SD
1 - Control (no antioxidant)	230.00 ± 1.92
2 - 6.5% proanthocyanidin	287.86 ± 1.55
3 - Cashew nut shell liquid	261.00 ± 1.85
4 - 10% Lycopene	359.00 ± 1.73

**Figure 1:**
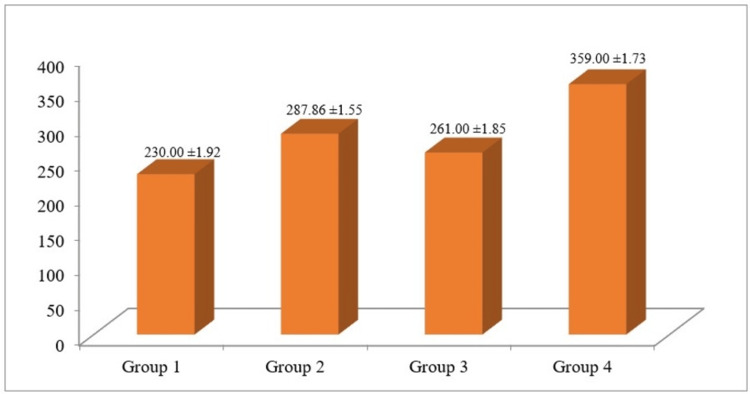
Evaluation and comparison of different antioxidants on the dentinal tubule penetrability of resin-based sealer.

Statistical analysis using one-way ANOVA followed by Tukey’s post hoc test revealed a statistically significant difference (p < 0.05) among the groups. Group 4 (lycopene) demonstrated significantly greater dentinal tubule penetration (359.00 ± 1.73 µm) compared to all other groups (p < 0.05). Conversely, group 1 (control) exhibited the lowest penetration depth (230.00 ± 1.92 μm), which was significantly lower than that of the antioxidant-treated groups (p < 0.05). No statistically significant difference was observed between group 2 (proanthocyanidin) and group 3 (CNSL) (Table 2, Figure 1).

**Figure 2:**
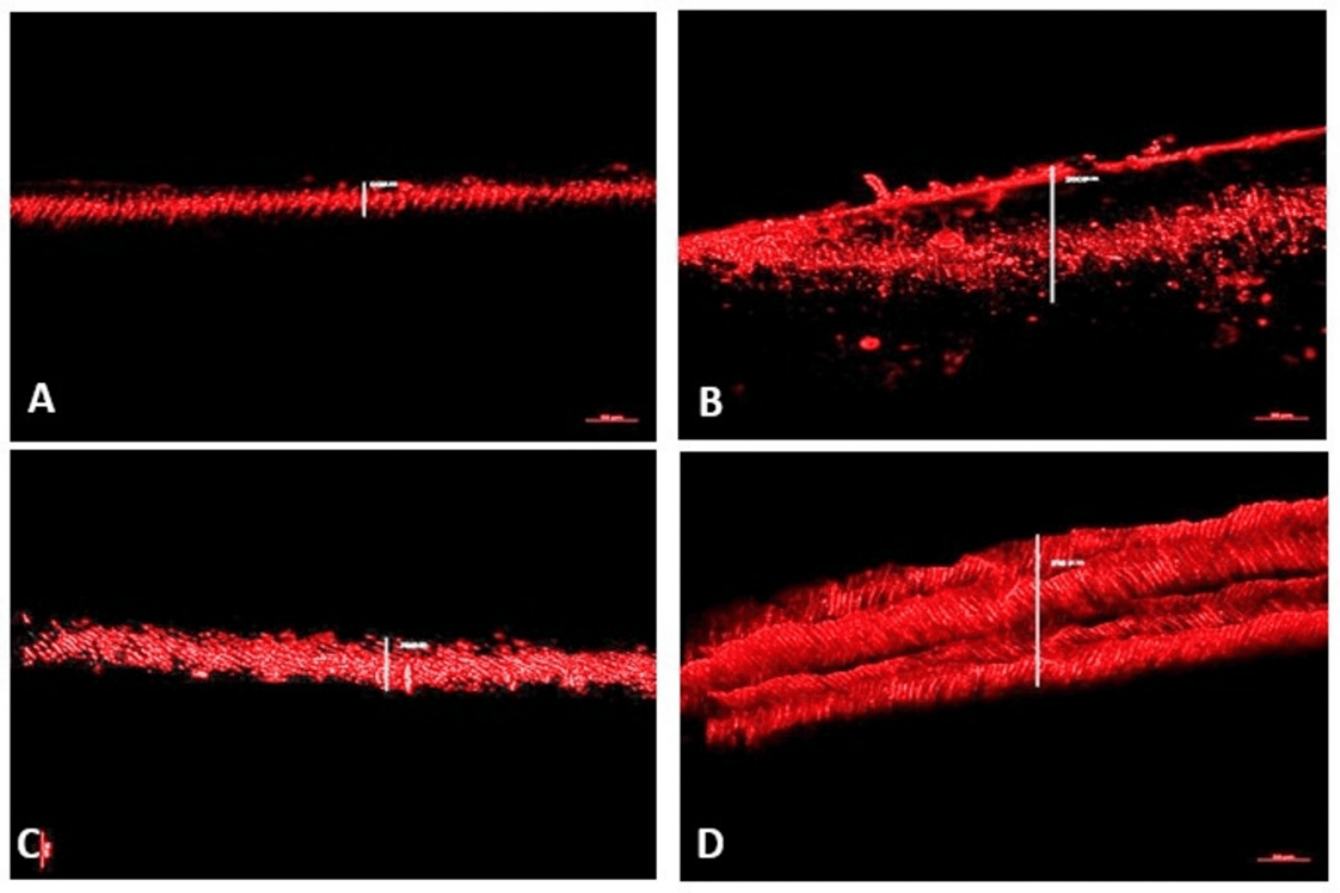
Confocal laser microscopy evaluation of samples. (A) Penetration depth of group 1 - control group with no antioxidants. (B) Penetration depth of group 2 - proanthocyanidin. (C) Penetration depth of group 3 - cashew nut shell liquid. (D) Penetration depth of group 4 - lycopene.

CLSM images demonstrated distinct patterns of sealer penetration along the root canal walls among the experimental groups. The depth and uniformity of sealer penetration into dentinal tubules varied with the type of antioxidant treatment employed. The control group (Figure 2A) and the CNSL-treated group (Figure 2C) exhibited minimal sealer penetration, indicating limited permeability of the dentinal tubules and suboptimal interfacial adaptation. In contrast, Group 4 specimens (Figure 2D) revealed the greatest depth and intensity of sealer penetration, suggesting enhanced dentin surface reactivity and improved sealer infiltration. This was followed by Group 2 (Figure 2B), which also showed appreciable tubule penetration but to a lesser extent than Group 4. These findings indicate that specific antioxidant treatments can significantly influence the intratubular diffusion characteristics of root canal sealers.

Post hoc analysis using multiple pairwise comparisons revealed a statistically significant difference in mean sealer penetration among all the experimental groups (p < 0.05). The comparison demonstrated that group 4 (10% lycopene) exhibited the highest mean sealer penetration depth, followed by group 2 (6.5% proanthocyanidin) and group 3 (CNSL). The control group (group 1) showed the lowest mean penetration values. All intergroup comparisons showed statistically significant differences (p = 0.001), indicating that the application of antioxidants enhanced the sealer’s dentinal tubule penetrability to varying degrees, with lycopene producing the most pronounced effect (Table 3).

**Table 3:** Post hoc analysis. Post hoc analysis showing comparison within groups revealing a significant difference between all the groups. Significantly higher values were reported in group 4, followed by group 2 and group 3, and the least in group 1.

	Group 1		Group 2		Group 3		Group 4	
	Mean difference	P-value	Mean difference	P-value	Mean difference	P-value	Mean difference	P-value
Group 1	-		-57.866	0.001*	-31.00	0.001*	-129.00	0.001*
Group 2	57.866	0.001*	-		26.866	0.001*	-71.13	0.001*
Group 3	31.00	0.001*	-26.866	0.001*	-		-98.00	0.001*
Group 4	129.00	0.001*	71.13	0.001*	98.00	0.001*	-	

## Discussion

The present study demonstrates the novel use of lycopene, proanthocyanidin, and CNSL as antioxidant agents to enhance resin sealer penetration. The randomized in vitro design and use of confocal microscopy provide robust and reproducible evidence.

A critical step of root canal treatment involves the proper use of various irrigants, mainly NaOCl. NaOCl dissolves the organic content of the smear layer and possesses strong antimicrobial properties, making it an ideal irrigant in endodontics [6]. However, NaOCl can also damage collagen in dentin by breaking down the hydrogen bonds between collagen fibrils, resulting in the collapse of the collagen network. This collapse may reduce the resin monomer’s penetrability into dentinal tubules. Moreover, NaOCl can interfere with resin polymerization because of the residual oxygen it leaves on the surface [7]. This residual oxygen prevents adequate bonding of resin-based sealers to dentin. To counter these negative effects, antioxidants have been recommended, as these agents can neutralize the free oxygen trapped in dentinal tubules and enhance cross-linking of the collagen network by forming new hydrogen bonds between collagen fibers [8].

In the current study, AH Plus sealer, an epoxy resin-based sealer, was used. According to a recent study, AH Plus sealer exhibits good dimensional stability, strong adhesion to dentin, low solubility, and high radiopacity [9].

CLSM was used to evaluate the depth of sealer penetration, as it offers several advantages over conventional optical microscopy and scanning electron microscopy. The benefits of using CLSM include control over the depth of field, reduction of background information away from the focal plane, and the ability to assemble multiple optical sections, even from thick specimens [10]. According to Elliot et al. (2020), when using CLSM, artifacts can be practically excluded [11]. Moreover, CLSM software can generate three-dimensional reconstructions of dentinal tubules, allowing accurate calculation of mean penetration depth, as demonstrated by Ordinola-Zapata et al. (2009) [12]. To improve sealer visibility within dentinal tubules, Rhodamine B dye was incorporated as a fluorescent indicator, as it enhances contrast and enables the sealer to be visualized under CLSM [13]. Thota et al. (2017) further reported that Rhodamine B dye does not alter the physical properties of the sealer [14].

Among the four experimental groups, the 10% lycopene group (group 4) exhibited the greatest mean sealer penetration (359.00 ± 1.73 µm), indicating superior efficacy in neutralizing residual oxygen and enhancing resin sealer adaptation to dentin. This was followed by the 6.5% proanthocyanidin group (group 2) (287.86 ± 1.55 µm) and the CSNL group (group 3) (261.00 ± 1.85 µm), both of which demonstrated significantly higher penetration than the control (group 1) (230.00 ± 1.92 µm). The comparable performance of proanthocyanidin and CSNL may reflect similarities in their antioxidant capacities, though variations in their molecular composition and diffusion potential could also influence their interaction with dentin substrates.

Group 4 (10% lycopene) demonstrated the highest mean depth of sealer penetration (359.00 ± 1.73 µm), indicating superior interaction between the sealer and dentinal substrate. Lycopene, a highly lipophilic carotenoid, exhibits strong free radical-scavenging activity and a low-viscosity profile, which may enhance its ability to diffuse through dentinal tubules and neutralize residual oxygen species. This property likely contributes to improved resin infiltration and interfacial adaptation. Verma et al. (2024) [15] reported that the reduced viscosity and favorable wetting characteristics of lycopene facilitate deeper sealer penetration within the dentinal microstructure. Similarly, Gupta et al. (2022) [16] demonstrated that lycopene’s potent antioxidant activity, mediated through its capacity to neutralize reactive oxygen species, can enhance the bonding interface and overall sealer penetration depth. Collectively, these findings support lycopene’s efficacy as an effective dentin pretreatment agent for optimizing sealer adaptation.

In contrast, group 1 (control, no antioxidant) exhibited the least dentinal tubule penetration (230.00 ±1.92 µm) compared with the antioxidant-treated groups. Cai et al. (2023) reported that NaOCl degrades collagen fibrils within dentinal tubules, compromising their structure [17]. In the absence of antioxidants, collagen fibrils remain collapsed, which may have limited the resin’s penetrability. Additionally, de Oliveira et al. (2022) demonstrated that NaOCl reacts with dentinal moisture to release nascent oxygen, which diffuses deep into tubules and inhibits resin polymerization [18], likely contributing to the reduced penetrability seen in the control group.

Intergroup comparisons revealed that group 2 (proanthocyanidin) achieved greater dentinal tubule penetration (287.86 ± 1.55 µm) than group 3 (CNSL) (261.00 ± 1.85 µm). Proanthocyanidins are hydrophilic compounds, and according to Qian et al. (2022), hydrophilic substances interact readily with the water present in dentin [19]. In contrast, CNSL is hydrophobic, leading to poor wetting and decreased adhesion of the root canal sealer, as supported by Tummala et al. (2012) [20].

Furthermore, Kalra (2013) reported that proanthocyanidins have a low molecular weight due to the presence of flavonoids such as catechin and epicatechin, facilitating deeper diffusion and oxygen scavenging within dentinal tubules [21]. Conversely, CNSL contains large molecules such as anacardic acid, which may hinder diffusion. Ike et al. (2021) found that the large molecular size of CNSL limits its ability to remove nascent oxygen, reducing sealer penetration [22]. Therefore, the CNSL-pretreated group showed lower penetrability than the other experimental groups.

Overall, the findings suggest that antioxidants such as lycopene, proanthocyanidins, and CNSL can improve root canal sealer penetration into dentinal tubules. Among these, lycopene demonstrated the greatest efficacy, making it a promising adjunct for enhancing the sealing ability of resin-based sealers and potentially improving long-term clinical outcomes. However, further in vivo and clinical studies are required to confirm the sustained effects of these antioxidants in endodontic therapy.

## Conclusions

Within the limitations of the present in vitro study, it can be concluded that lycopene, owing to its potent antioxidant capacity and reduced viscosity, demonstrated the greatest ability to enhance sealer penetration within dentinal tubules. This was followed by proanthocyanidin and CNSL. The findings suggest that the use of antioxidants as a final irrigant or pretreatment agent can significantly improve the depth of resin-based sealer penetration, thereby contributing to the formation of a more effective, fluid-tight hermetic seal. Consequently, incorporating antioxidant agents such as lycopene before obturation may improve sealer penetration and interfacial adaptation under in vitro conditions, suggesting a potential role for such agents in optimizing root canal sealing. However, further in vivo studies are required to substantiate their clinical applicability and long-term benefits.
